# Dynamic Light Scattering Based Microrheology of End-Functionalised Triblock Copolymer Solutions

**DOI:** 10.3390/polym15030481

**Published:** 2023-01-17

**Authors:** Ren Liu, Alessio Caciagli, Jiaming Yu, Xiaoying Tang, Rini Ghosh, Erika Eiser

**Affiliations:** 1 Cavendish Laboratory, University of Cambridge, Cambridge CB3 0HE, UK; 2 PoreLab, Department of Physics, Norwegian University of Science and Technology, N-7491 Trondheim, Norway

**Keywords:** competing self-assembly, triblock-copolymer, DNA, dynamic light scattering, microrheology, transient hydrogels, telechelic polymers

## Abstract

Nano-sized particles functionalised with short single-stranded (ss)DNAs can act as detectors of complementary DNA strands. Here we consider tri-block-copolymer-based, self-assembling DNA-coated nanoparticles. The copolymers are chemically linked to the DNA strands via azide (N${}_{3}$) groups. The micelles aggregate when they are linked with complementary ssDNA. The advantage of such block-copolymer-based systems is that they are easy to make. Here we show that DNA functionalisation results in inter-micellar attraction, but that N${}_{3}$-groups that have not reacted with the DNA detector strands also change the phase behaviour of the tri-block polymer solution. We studied the triblock copolymer, Pluronic^®^ F108, which forms spherical micelles in aqueous solutions upon heating. We find that the triblock chains ending with either an N${}_{3}$ or N${}_{3}$-DNA complex show a dramatic change in phase behaviour. In particular, the N${}_{3}$-functionalisation causes the chain ends to cluster below the critical micelle temperature (CMT) of pure F108, forming flower-micelles with the N${}_{3}$-groups at the core, while the PPO groups are exposed to the solvent. Above the CMT, we see an inversion with the PPO chains forming the micellar core, while the N${}_{3}$-groups are now aggregating on the periphery, inducing an attraction between the micelles. Our results demonstrate that, due to the two competing self-assembling mechanisms, the system can form transient hydrogels.

## 1. Introduction

The self-assembly of block copolymers into microphase-separated structures is a research topic of high relevance in polymer science and various practical applications in nanoscience and biotechnology [1,2,3]. Depending on their block-size ratio, copolymers can self-assemble into shapes such as spheres, cylinders and bilayers [4], but they are not suitable as building blocks for higher-order assemblies. Recently, patchy micelles have been prepared based on directed self-assembly of ABC type triblock copolymers, forming multicompartment micelles to achieve superstructures [5]. This has greatly expanded the concept of ‘patchy’ colloids, as the building subunit can now be tuned from soft to hard matter, and hybrid systems can be designed [6], bringing a whole new dimension to the use of ‘patchy’ colloids in the rational design of hierarchical super-structures [7,8,9].

DNA hybridisation is a major player in the realisation of functional and patchy particles with highly selective interactions [10,11]. Due to its thermo-reversibility and high specificity, it has been extensively employed to drive and direct colloidal self-assembly via temperature control [12,13,14]. Both ‘hard’ and ‘soft’ colloids have been successfully functionalised with DNA [15,16,17,18,19,20,21,22]. Their extension to polymeric micelles has, however, not been attempted yet. Recent advances have shown the possibility of attaching DNA to PEG-PPO-PEG triblock copolymers [23,24]. These copolymers, commercially known as Synperonic (manufactured by Croda), Pluronics^®^ (BASF) or Poloxamers (ICI), have been successfully used in several applied areas such as particle synthesis [25], emulsion formulation [26], cosmetics [27] and coatings [28]. The temperature-responsive, non-toxic nature of Pluronics^®^ makes them ‘smart’ polymers that actively respond to changes in the surrounding environment, rendering them excellent candidates for applications in drug delivery [29,30]. By combining their intrinsic thermal response to the specific and thermo-reversible DNA hybridisation mechanism, novel structures with exotic phase diagrams can be envisioned. In particular, it could be possible to fine-tune the rheological properties of the underlying system in a similar way to DNA-based hydrogels [31,32,33] but with the advantages of better scaleability. This opens the possibility for a large number of applications related to biomedicine and drug delivery, in which fine control of the materials’ mechanical properties is required.

Here we present a first study on the aggregation and phase behaviour of the N${}_{3}$ and N${}_{3}$-DNA functionalised triblock copolymer F108, which is known to form spherical micelles upon heating. We used a simple protocol to first attach the azide groups to the free PEO-ends of F108, of which a fraction was reacted to single-stranded DNA oligonucleotides via click-chemistry. The structural and rheological properties of the aqueous solutions of these systems were then studied as function of concentration and temperature via Dynamic Light Scattering (DLS) and DLS-based passive microrheology. The methodology for the latter is based on DLS in the single scattering limit, enabling us to measure the viscoelasticity of our system over a frequency range of 1–10${}^{5}\;s^{-1}$. Our results show an unexpected phase behaviour that emerged from the presence of free PEO-azide ends that showed attractive, non-specific interactions between each other, which became stronger with concentration and temperature, enriching the overall Pluronics phase diagram.

## 2. Materials and Methods

Synperonic F108 (PEG−poly(propylene oxide) (PPO)−PEG), dichloromethane (DCM), triethylamine, 4-Toluenesulfonyl chloride (TsCl), diethyl ether, NaN${}_{3}$ and solvents (purity >95%) were purchased from Sigma-Aldrich. Here we will refer to the triblock-copolymer simply as Pluronic F108 or F108 alone. The DNA single strands (ssDNA) were obtained from Integrated DNA Technologies (IDT).

### 2.1. Sample Preparation

#### 2.1.1. Functionalisation of F108 with Azide Groups (N${}_{3}$-PEG-PPO-PEG-N${}_{3}$)

We covalently attached N${}_{3}$-groups to the PEO chain ends of F108, following a protocol described by Caciagli et al. [23]. This protocal was adapted from a method introduced by Oh et al. [34]. The yield of this azide-functionalisation was > 90%. The final product, F108-N${}_{3}$, was dried under vacuum and stored in a freezer.

#### 2.1.2. Dibenzylcyclooctane (DBCO)-DNA Preparation

The complementary DNA strands used were: amine-5${}^{\prime }$-TTT TTT TTT TTT TTT GGT GCT GCG-3${}^{\prime }$, called *A* and *A${}^{\prime }$*, amine-5${}^{\prime }$-TTT TTT TTT TTT TTT CGC AGC ACC-3${}^{\prime }$. Both have a 15 thymine (T) long, non-binding spacer. The molecular weight of the DNA strands is $M_{w}$ = 7512 ${\mathrm{gmol}}^{-1}$, and the melting temperature, at which half of all hydrogen-bonds are broken, was found to be $T_{m}∼48{\;}^{{}^{\circ}}$C, close to the one based on the nearest neighbour model by SanatLucia [35]. Here, we followed the protocol of amine-to-dibenzylcyclooctane (DBCO) functionalisation described by Zupkauskas et al. [36]. FTIR and zeta potential measurements, shown in the PhD thesis of Zupkauskas suggest that the PEO-azide groups are not charged. The DBCO-DNA was then upconcentrated using a centrifugal filter (Amicon Ultra-0.5 Centrifugal Filter, MWCO = 3000 ${\mathrm{gmol}}^{-1}$) to a maximum concentration of 5 ${\mathrm{mmolL}}^{-1}$. The DBCO-DNA was kept frozen in 10 mM phosphate buffer (PB) at 0.05 mmolL${}^{-1}$ until needed.

#### 2.1.3. DNA Functionalised F108 Preparation

Following a protocol described by Caciagli et al. [23], 100 μL F108-N${}_{3}$ was mixed with 500 μL of DBCO-DNA and 400 μL of 10 mM phosphate buffer. The solution was reacted at 65 ${}^{{}^{\circ}}$C via a strain-promoted alkyne-azide click reaction (SPAAC), while vigorously shaking for 24 h. No further washing or centrifugation of the resultant solution was required. Beside the challenges in the realisation (which would require dialysis or centrifugation of composites with similar molecular weight) we reasoned that the SPAAC reaction achieved very high yields (≥ 90%) and we performed the functionalisation step in small volumes. Hence, we assumed that most ssDNA was covalently linked to F108-N${}_{3}$ ends and only a negligible amount of ssDNA remained free in solution. All F108-N${}_{3}$ samples, presented here, were prepared with a 1:1 ratio of F108-*A*-DNA and F108-*A${}^{\prime }$*-DNA, unless specified otherwise.

### 2.2. F108 Phase Diagram Study

In this study, 2 cm wide glass vials were used for the sample preparation. F108 flakes were dissolved in 10 mM PB buffer with 100 mM NaCl at 4 ${}^{{}^{\circ}}$C, for which F108 is fully soluble in water. Final concentrations of 5–25 % w/v F108 solutions were prepared and kept refrigerated while equilibrating for 3 days. Visual observations at different temperatures were carried out by immersing sealed samples in either a water or oil bath, for temperatures varying between 5 and 85 ${}^{{}^{\circ}}$C. Samples were equilibrated for at least 30 min at each temperature before a visual inspection was performed to determine their respective phase state.

### 2.3. Dynamic Light Scattering

DLS measurements were performed using a Malvern Zetasizer, Nano ZS (633 nm laser). Low-volume measurements were preformed using 40 μL microcuvettes (Malvern ZEN0040). Samples were equilibrated for 20 min at each temperature to ensure thermal and hydrodynamic equilibrium prior to each run. The duration of each run was adjusted automatically according to the estimated relaxation time of the respective sample (i.e., the time required for $g^{\left(2\right)}\left(\tau \right)$ to decay to 1) and varied between 100 s and 30 min. An average over three consecutive runs constitutes a measurement.

### 2.4. DLS-Based Microrheology

We employed 230 nm large polystyrene spheres coated with polyethylene glycol (PS-PEG) as tracer particles, which were provided by Cambridge Bespoke Colloids, UK. Sample evaporation was prevented by adding a thin layer of silicone oil (50 cSt) in the cuvette on top of the sample (measurements were unaffected by this addition). To ensure the detection of only single-scattering events, the setup was operated in a non-invasive backscattering (NIBS) mode at a scattering angle of 173${}^{{}^{\circ}}$. In this setting, optimal scattering was achieved for a particle volume fraction of 0.03%, ensuring that the probe scattering dominated over direct scattering from the sample, accounting for over 90% of the signal. We verified that the colloids did not interact with each other by performing the DLS measurements under the same experimental conditions, but different concentrations [37,38].

An in-house developed constrained regularisation (CONTIN) method was used to analyse the measured scattering intensities [38]. The raw intensity-autocorrelation functions $g^{\left(2\right)}\left(q,\tau \right)$) were normalised and then converted into electric-field autocorrelation functions $g^{\left(1\right)}\left(q,\tau \right)=g^{\left(1\right)}\left(\tau \right)$ with Matlab routines. After fitting the $g^{\left(1\right)}\left(\tau \right)$ curves using another in-house routine, we extracted the corresponding mean-squared displacements (MSDs). From these MSDs we extracted the viscosities of our samples in the zero-shear limit. Further, by Fourier transforming the MSDs we also obtained the elastic and viscous moduli, $G^{\prime }\left(\omega \right)$ and $G^{\prime \prime }\left(\omega \right)$, using the generalised Stokes–Einstein relation [37]. A detailed explanation of our in-house developed Matlab routines will be presented in a separate paper, where they will be made available.

## 3. Results

### 3.1. Design and Phase Diagram of the System

**Figure 1:**
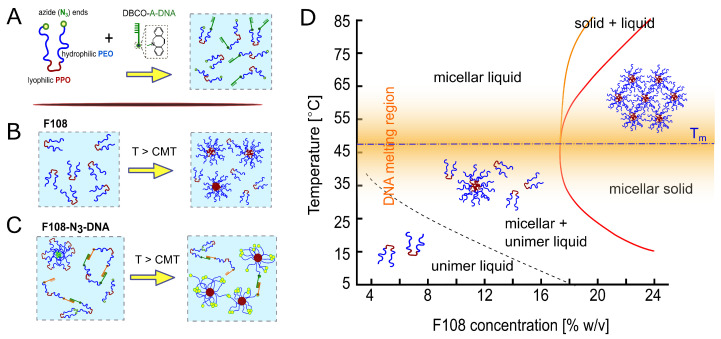
(**A**) Schematic of F108 with azide-functionalised PEG-ends. N${}_{3}$-groups were reacted to DBCO-*A*-DNA with a 10:1 molar fraction, using click-chemistry. Below the CMT, F108-N${}_{3}$-*A/A${}^{\prime }$* unimers are well dispersed in water. (**B**) Non-functional F108 chains undergo a microphase separation from unimers to spherical micelles above the CMT, approximated by the dashed line. (**C**) Below the CMT, chains with complementary DNA can bind forming rings or chains while the remaining F108-N${}_{3}$-chains form transient flower micelles. Above the CMT, the unimers form standard micelles in addition to maintaining the *AA${}^{\prime }$* bonds for $T<T_{m}∼48{\;}^{{}^{\circ}}$C. (dash-dotted line); the width of the DNA melting region is indicated by the orange shaded region in the phase diagram. (**D**) Experimental phase diagram of F108 in buffer solution containing 100 mM NaCl, based on optical observations. The red line marks the transition to a single micellar solid phase while the region between the orange and red line indicates a micellar solid coexisting with a micellar liquid.

The phase diagram of non-functionalised F108 in aqueous solutions containing 100 mM NaCl is presented in Figure 1. F108 is a symmetric triblock copolymer of the form (EO)${}_{147}$-(PO)${}_{56}$-(EO)${}_{147}$ with an average molecular weight of $M_{w}$ = 14,600 ${\mathrm{gmol}}^{-1}$. It belongs to the hydrophilic spectrum of the Pluronics family [39]. Because of their hydrophilic-to-hydrophobic size ratio, F108 chains can only form spherical micelles above a critical micelle temperature (CMT) [40,41]; the latter depends weakly on the F108 concentration and can be lowered substantially by adding salt [42,43,44].

Aqueous F108, F108-N${}_{3}$ and F108-N${}_{3}$-DNA solutions are transparent. Therefore, the transition from unimer to micellar liquid can only be determined either via Small Angle X-ray Scattering (SAXS), rheology [43,45] or micro-DSC [44]. The transition from a micellar liquid to micellar solid is marked by a clear liquid to solid transition with a very narrow coexistence region. Hence, we determined the phase diagram of F108 solutions using visual inspections of sample tubes holding polymer concentrations between 5 and 25 % w/v, with finer concentration spacings at around 18 % w/v. The visual inspections were made in 2–4 ${}^{{}^{\circ}}$C heating steps between room temperature and 85 ${}^{{}^{\circ}}$C. The resulting phase diagram is shown in Figure 1). Here the lines represent a guide to the eye to respective phase transitions. Similar to earlier work by Diat et al. [42], solutions containing F108 concentrations below 18 % w/v remained homogeneously fluid up to 85 ${}^{{}^{\circ}}$C. Above ∼18 % w/v the samples showed a clear fluid to solid transition, which we observed by carefully tumbling the sample. Like in Ref. [40], the entire samples solidified upon heating (red line in Figure 1) forming a micellar crystal with face-centred cubic structure, which was verified with SAXS (these data will be presented in a separate publication; see also PhD thesis of R. Liu [46]). However, upon further heating, we observed partial melting (region between orange and red line in Figure 1), which we attribute to chain-length polydispersity [47,48]. It should be noted that Pluronics are industrial products that show large batch-to-batch variations in the CMT and liquid to solid transition boundaries. Nevertheless, the shape of the phase diagram and the micellar structure of the fluid and crystalline phases remain the same for a Pluronic material with a given name.

ssDNA was attached to the free PEO ends by first functionalising them with a N${}_{3}$-group with a yield above 90 % (Figure 1A). Subsequently, DBCO-functionalised ssDNA strands were attached to the N${}_{3}$ ends of the block copolymers via a strain-promoted alkyne-azide click reaction (SPAAC). F108-*A*-DNA and F108-*A${}^{\prime }$*-DNA solutions were prepared separately and then mixed in 1:1 ratios to obtain the final concentrations. In the following we refer to these mixtures as F108-N${}_{3}$-*AA${}^{\prime }$* solutions. We did not wash or centrifuge the resultant solutions because of the high yields of the click reaction. Because of the high cost of using large ssDNA concentrations, we functionalised only a fraction of the F108-N${}_{3}$ ends with ssDNA; here we refer to 10:1 molar ratios simply as F108-N${}_{3}$-*AA${}^{\prime }$* samples, unless stated otherwise. Therefore, we performed mainly DLS studies of these samples in the dilute and semi-dilute liquid state, as these measurements require only small volumina. A simulation study on the detailed phase diagram of pure F108 and F108-*AA${}^{\prime }$* solutions, in which all ends are DNA-functionalised, can be found in the PhD thesis of J. Yu [49], which will be presented in a separate publication.

### 3.2. DLS and DLS-Microrheology Study of Semi-Dilute Solutions

To study the micellar sizes of our system as function of temperature and the addition of DNA to a fraction of the Pluronic chains we first investigated the properties of 5 % w/v F108 and F108-N${}_{3}$-*AA${}^{\prime }$* solutions in 100 mM NaCl. The added salt ensured the equilibrium hybridisation (binding) of the complementary DNA strands [10]. We estimated that the added NaCl lowers the CMT by around 1 ${}^{{}^{\circ}}$C, and thus could be neglected [43,50]. At high enough temperatures, where all F108-unimers are known to form spherical micelles we estimated their volume fraction to be ϕ∼ 0.03. Hence, micelle–micelle interactions can be neglected, allowing us to measure the micellar radius and eventual differences between the two systems. The raw auto-correlation curves $g^{\left(1\right)}\left(\tau \right)$ obtained by DLS showed a single-exponential decay for both systems in the range of 25 to 60 ${}^{{}^{\circ}}$C. We used a cumulant analysis method [38] to extract the micellar hydrodynamic diameter $D_{H}$ and polydispersity index by fitting the short-time portion of the auto-correlation function as

(1) $$\log\left(g^{\left(1\right)}\left(\tau \right)\right)=-\Gamma \left(\tau -\frac{\mu_{2}}{2}\tau^{2}\right).$$

**Figure 2:**
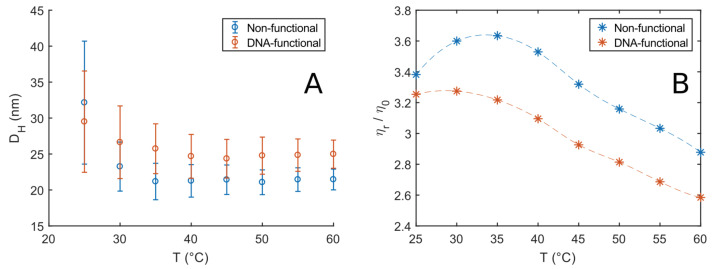
(**A**) Hydrodynamic diameter $D_{H}$ of 5% w/v F108 and F108-N${}_{3}$-*AA${}^{\prime }$* solutions estimated by cumulant analysis at different temperatures. The error bars represent the polydispersity index (PDI). (**B**) Relative viscosities, normalised by the solvent viscosity $\eta_{0}$ measured as function of temperature for the solutions in (**A**) obtained via DLS microrheology: The $\eta_{r}$ values were obtained from the long-time frequency region of the MSDs.

Here, $\Gamma =q^{2}D$ is the decay rate, $\mu_{2}$ the second cumulant and $\mu_{2}/\Gamma^{2}$ is the polydispersity index (PDI). Γ and the PDI are fitting parameters, *q* is the scattering vector and *D* the diffusion coefficient of the scattering micelles, which was obtained via the Stokes–Einstein relation

(2) $$D_{H}=\frac{q^{2}k_{B}T}{3\pi \eta_{s}\Gamma },$$

with the solvent viscosity $\eta_{s}$. The latter was taken to be that of H${}_{2}$O, as listed in the NIST database [51]. Figure 2A shows the calculated hydrodynamic diameters of 5 % w/v solutions of F108 and F108-N${}_{3}$-*AA${}^{\prime }$*; the error bars represent the PDI. Both solutions show a larger hydrodynamic size at temperatures close to the CMT, which is at roughly 23 ${}^{{}^{\circ}}$C for F108 at this concentration. Such a behaviour is typical for Pluronic solutions [52,53,54]. It is attributed to the coexistence of unimers and micelles rapidly forming and disintegrating in the transition regime around the CMT and above. Outside the transition regime ($T>35{\;}^{{}^{\circ}}$C) the hydrodynamic diameter is independent of temperature for both solutions. These findings suggest that the transition or rather coexistence region containing unimers and micelles is about 10–15 ${}^{{}^{\circ}}$C wide, in agreement with previous reports [54,55]. Conversely, the scattering intensity plateaus around a maximum value for $T>35{\;}^{{}^{\circ}}$C, which indicates that the number of micelles in the systems remains constant. F108 displayed an average $D_{H}$ of 22 nm, similar to earlier findings [42], while the F108-N${}_{3}$-*AA${}^{\prime }$* DNA solutions showed a slightly larger micellar diameter of $D_{H}∼25$ nm. The size increase is most likely due to the presence of the ssDNA as the hydrophobic N${}_{3}$-end groups are considerably smaller. The persistence length of ssDNA is of the order of 1 nm, corresponding to about 3 bases [56]: Our ssDNAs are 24 bases long, and have a fully stretched length of about 8 nm; however, because they are in a good solvent, they will coil up, leading to a size that roughly matches the observed increase in the micelle diameter. Moreover, the F108-N${}_{3}$-*AA${}^{\prime }$* solutions show a slower decay to the plateau value of $D_{H}$ than F108, becoming temperature-independent around $T∼45{\;}^{{}^{\circ}}$C. This increase in the width of the transition region is easily understood: While water is a good solvent for the PPO middle-block of the non-ionic F108 at low temperatures it becomes an increasingly less good solvent as *T* increases. The addition of the negatively charged ssDNA will, however, increase the solubility of the F108 chains holding one or two DNA strands and thus shift all phase boundaries of the system to higher temperatures. Moreover, we do not observe any effect due to the DNA-binding below $T_{m}$ or binding between the free PEO-N${}_{3}$-end groups at these low concentrations.

To further elucidate these observations, we performed DLS microrheology using 230 nm large probe particles, as explained in the experimental section. At 5 % w/v, both systems show little to no elasticity and can be characterised by their zero-frequency viscosity. From the auto-correlation function

(3) $$g^{\left(1\right)}\left(\tau \right)=\exp\left(-\frac{1}{6}q^{2}\langle \Delta r^{2}\left(\tau \right)\rangle \right),$$

We obtained the MSDs ($\langle \Delta r^{2}\left(\tau \right)\rangle$) as function of temperature, from which we extracted the viscosities. Figure 2B shows the viscosity curves of the two systems relative to the solvent viscosity. The trend observed for the F108 solutions is in agreement with measurements on similar systems [43,50]. In particular, the initial upward trend in viscosity is due to the constant increase of the number of micelles in the system in the transition region. This translates to larger micellar volume fractions and a consequent increase in viscosity according to Einstein’s equation

(4) $$\eta =\eta_{0}\left(1+2.5\phi_{eff}\right).$$

Here we considered the micelles to behave as spherical particles with an effective volume fraction $\phi_{eff}$ [43].

The peak at around $T∼35{\;}^{{}^{\circ}}$C roughly indicates the end of the transition region for the non-functional F108 solutions, where all unimers are converted into micelles. After this point, the viscosity shows a linear decrease with increasing temperature reflecting the decreasing viscosity of the aqueous solvent. A similar but less pronounced trend was observed for the F108-N${}_{3}$-*AA${}^{\prime }$* solutions.

### 3.3. DLS and Microrheology Study of Semi-Concentrated Solutions

**Figure 3:**
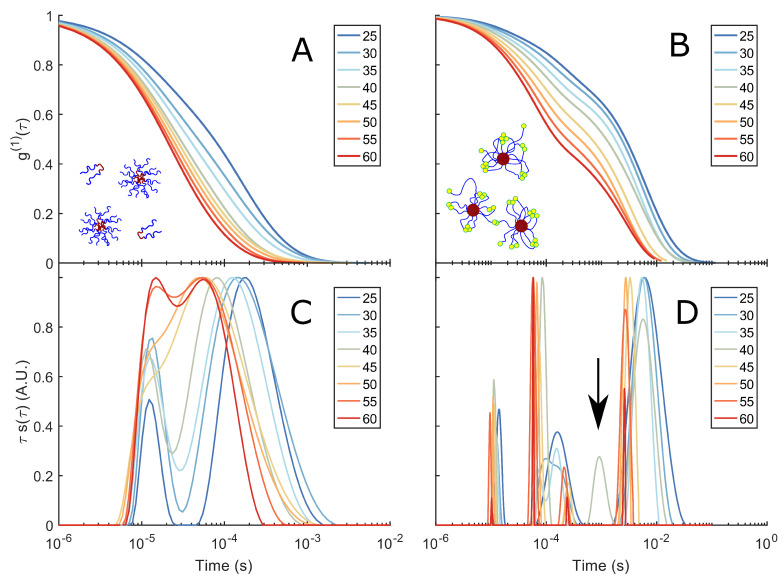
DLS spectra and corresponding relaxation times: (**A**) $g^{\left(1\right)}\left(\tau \right)$ of a 10% w/v F108 solution in 100 mM NaCl measured in DLS as function of temperature. (**C**) Corresponding relaxation–time distributions s(τ), obtained by constrained regularisation fitting of the experimental $g^{\left(1\right)}\left(\tau \right)$ using an in-house routine [37]. The vertical axis τs(τ) provides an equal area representation. The curves have been normalised to the highest peak of s(τ) to facilitate the comparison between different temperatures. (**B**) $g^{\left(1\right)}\left(\tau \right)$ of a 10 % w/v F108-N${}_{3}$-*AA${}^{\prime }$* solution in 100 mM NaCl and (**D**) the corresponding distributions of relaxation times. The arrow indicates the small peak at $\tau ∼10^{-3}$ s for *T* = 40 °C.

When doubling the concentrations of F108 and F108-N${}_{3}$-*AA${}^{\prime }$* to 10 % w/v in 100 mM NaCl, the CMT of the F108 system is estimated to decrease to about 21 ${}^{{}^{\circ}}$C [50,57]. At this concentration, micelle–micelle interactions can occur even though the system appeared homogeneously liquid and transparent between 5 and 80 ${}^{{}^{\circ}}$C on visual inspection, which was supported by the decay of the correlation functions to zero. Figure 3 shows the raw autocorrelation curves $g^{\left(1\right)}\left(\tau \right)$ obtained by DLS for the F108 and F108-N${}_{3}$-*AA${}^{\prime }$* (1:10) solutions in a temperature range of 25–60 ${}^{{}^{\circ}}$C. The $g^{\left(1\right)}\left(\tau \right)$ curves were analysed using a constrained regularisation fitting-routine of the following form:

(5) $$g^{\left(1\right)}\left(\tau \right)=g_{0}+\int_{a}^{b}d\tau^{\prime }\;B\left(\tau^{\prime }\right)e^{-\tau /\tau^{\prime }}.$$

Plotting τs(τ) divided by the maximum of the individual relaxation times gives us the normalised relaxation–time curves in Figure 3C,D.

At 25 ${}^{{}^{\circ}}$C the F108 solution is in the micelle–unimer coexistence region and shows two characteristic decay times: One at $\tau_{1}\approx 1.2\times 10^{-5}$ s that is present at all temperatures studied and a longer relaxation time $\tau_{2}\approx 1.6\times 10^{-4}$ s with a tail up to $10^{-3}$ s. At first, one might associate these two relaxation times with the coexisting unimers and micelles. However, using Einstein’s relation between the MSD and the diffusion coefficient of the micelles and the radius of gyration of F108 unimers (∼5.4 nm [58]), we estimate them to be $1\times 10^{-5}$ s and $0.7\times 10^{-5}$ s. Hence, both relaxation times fall within $\tau_{1}$.

$\tau_{2}$ becomes a stretched exponential at higher temperatures and shifts towards faster decay times. This suggests the presence of small clusters at lower temperatures and their progressive disappearance with increasing temperature, leading to predominantly single micelles in solution. These findings are in contrast to extensive SAXS measurements by the group of Porte and others on non-functional F108 and F68, latter having the same hydrophobic to hydrophilic ratio but lower molecular weight [42,45,48,59]. In these studies, SAXS spectra taken for concentrations about 10 % w/v show a flat scattering spectrum in the unimer region (*T* < CMT) and a typical liquid ring in the micellar region (*T* > CMT). A cluster phase could not be discerned as these would only show at even lower scattering vectors, which were not accessible at the time. Static and dynamic scattering measurements by Brown et al. [53] on yet other Pluronic systems showed relaxation times for unimers and micelles similar to those we find, in addition to the signature of larger, disordered aggregates at longer relaxation times. This aggregation behaviour at lower temperatures indicates the presence of weak inter-unimer and inter-micellar interactions at these concentrations, hinting at the fact that PEO has a second upper critical solution point in addition to a lower critical solution point at around 100 ${}^{{}^{\circ}}$C [60,61,62] that may be also influenced by the PPO block. Indeed, when allowing the sample to settle in a glass tube for several hours and then tumbling it gently, one observes very weak Schlieren textures that disappear upon redispersion/homogenisation of the sample through vigorous stirring. Such behaviour was also observed by Wu et al. [43] and Patel et al. [50], in particular in saline solutions.

The 10 % w/v F108-N${}_{3}$-*AA${}^{\prime }$* solution delivered markedly different $g^{\left(1\right)}\left(\tau \right)$ curves. Analysis showed that in addition to the two relaxation times observed for the pure F108 samples, a third relaxation time $\tau_{3}\approx 10^{-2}$ s appears at 25 ${}^{{}^{\circ}}$C. Again we see a gradual transition of $\tau_{2}$ to a double-exponential decay with increasing temperatures, developing all together 4 distinct decay times at 60 ${}^{{}^{\circ}}$C. Only at around 40 ${}^{{}^{\circ}}$C the system showed an additional 5th peak at ∼$10^{-3}$ s. The presence of this peak is interesting, as it coincides roughly with the temperature at which all complementary DNA stands have hybridised and, thus, are possibly linking copolymers into small micellar chains. On increasing the temperature above $T_{m}=48{\;}^{{}^{\circ}}$C this 5th peak disappears. To understand the presence of very long relaxation times around $\tau_{3}$ that persisted at all temperatures studied, we need to keep in mind that a large fraction of the Pluronic chain ends azide groups that are known not to carry a net charge [63]. We hypothesised that these groups become increasingly hydrophobic at higher temperatures and concentrations. Therefore, the azide groups will tend to form flower micelles, meaning the F108 micelles must aggregate to fulfil this assembling drive, which is expressed in the long relaxation times. However, at high temperatures, thermal fluctuations will also break these azide–azide interactions; hence, the clusters must become smaller again, which we see in the red curves in Figure 3D.

**Figure 4:**
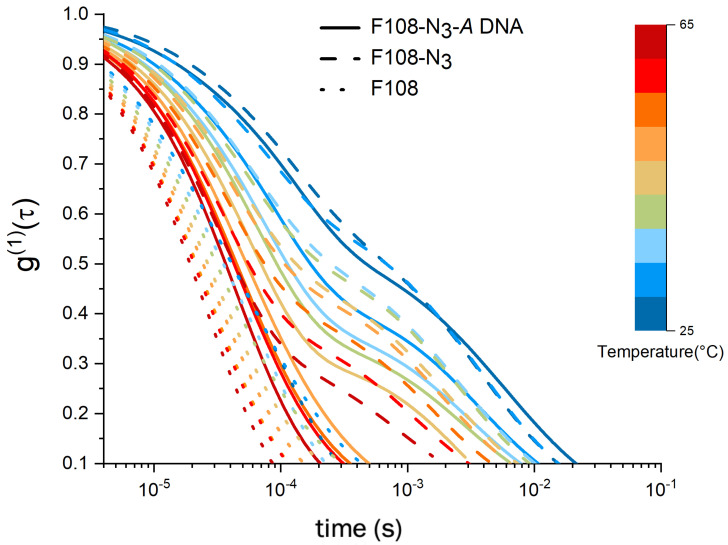
Comparison of $g^{\left(1\right)}\left(\tau \right)$ spectra of a 8.25 % w/v F108 with a F108-N${}_{3}$ and a F108-N${}_{3}$-DNA (10:1) solution containing only *A*-DNA such that no hybridisation can take place. All solutions were prepared in 100 mM NaCl and measured in DLS as function of temperature.

To this end we performed DLS studies on F108-N${}_{3}$ samples without the DNA attachment. In Figure 4, we compare 8.25 % w/v samples of F108-N${}_{3}$ with the pure F108 and F108-N${}_{3}$-DNA (10:1) carrying only *A*-DNA, not allowing for binding between PEO-chain ends. The electric field autocorrelation functions show a clear difference between the non-functionalised F108 and the one with azide and azide-DNA. In general, we observed that the $g^{\left(1\right)}\left(\tau \right)$ curves for F108 decay faster as the temperature increases, with no distinctly long relaxation time $\tau_{3}$. However, both F108-N${}_{3}$ and F108-N${}_{3}$-*A* samples display similar $\tau_{3}$ relaxations at 25 ${}^{{}^{\circ}}$C. The similarity between the autocorrelation functions of these two azide carrying systems gradually disappeared with increasing temperature. Above 40 ${}^{{}^{\circ}}$C, the F108-N${}_{3}$-*A* sample lost this $\tau_{3}$ relaxation and behaved more like the pure F108 sample. SAXS measurements of the F108-N${}_{3}$ system indeed revealed strong aggregation with eventual macroscopic phase separation into a polymer rich solid phase and a very fluid, polymer pure region above 70 ${}^{{}^{\circ}}$C and 16 % w/v. A detailed SAXS analysis of these phases will be published in a separate paper.

### 3.4. Microrheology of Concentrated Solutions

**Figure 5:**
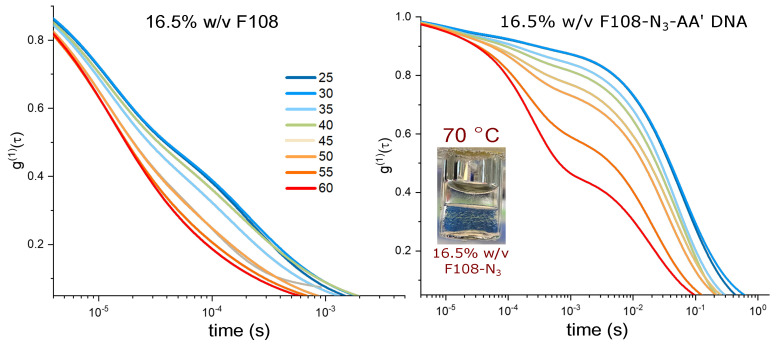
Comparison of $g^{\left(1\right)}\left(\tau \right)$ spectra of a 16.5 % w/v F108 and F108-N${}_{3}$-*AA${}^{\prime }$* (10:1) solutions. All solutions were prepared in 100 mM NaCl and measured in DLS as function of temperature. The inset shows a photograph of a phase separated F108-N${}_{3}$ solution heated to 70 ${}^{{}^{\circ}}$C.

A macroscopic phase separation in pure F108-N${}_{3}$ solutions appeared only at ≥70 ${}^{{}^{\circ}}$C for concentrations larger than 16 % w/v and started as an overall clouding of the sample. This clouding disappeared after holding the sample at this elevated temperature for about an hour, after which two clear phases separated by a flat interface emerged (inset in Figure 5). This phase separation was thermally reversible, showing no hysteresis, as long as heating and cooling were sufficiently slow. It should be noted that the F108 samples did not show any macroscopic phase separation below 18 % w/v.

Remarkably, the F108-N${}_{3}$-*AA${}^{\prime }$* solution also did not show such macroscopic phase separation. This is most likely due to the increased solubility provided by the DNA even though only a fraction of the F108-N${}_{3}$ chains had DNA attached. Nevertheless, the comparison of the autocorrelation functions for 16.5 % w/v, shown in Figure 5, reveals even longer relaxation times for the F108-N${}_{3}$-*AA${}^{\prime }$* solution, while those for the F108 solution do not show a drastic change, except that the overall bulk viscosity has increased, in agreement with previous measurements on the Pluronic F68 [43].

### 3.5. The Effect of Added Salt and Increased DNA Ratio

**Figure 6:**
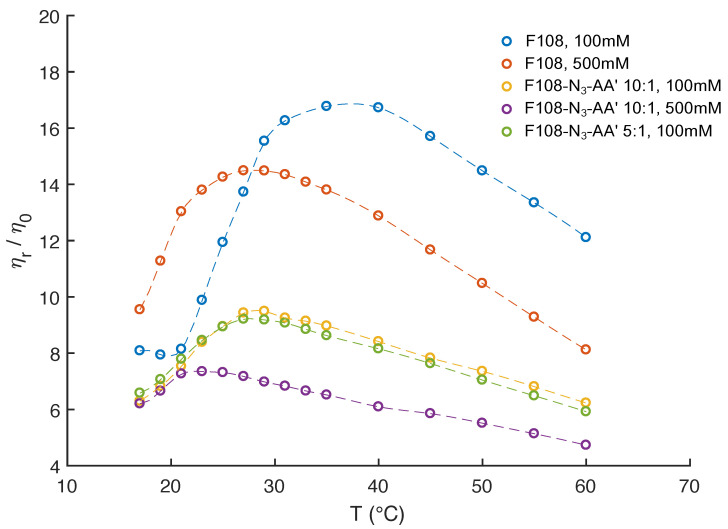
Relative viscosities of 10 % w/v F108, F108-N${}_{3}$-*AA${}^{\prime }$* with 10:1 and with 5:1 molar ratios as function of temperature and added NaCl, extracted from MSDs measured in DLS based microrheology.

The above observations demonstrate that the free azide ends on the F108 chains strongly influence their overall micellisation and aggregation behaviour. At the same time, functionalising the PEO-azide ends with DNA increases the solubility of the F108 chains in water, suppressing the macroscopic phase separation of the F108-N${}_{3}$ solutions at higher concentrations and temperatures. In this context, we also examined the effect of added salt on the pure F108 and F108-N${}_{3}$-*AA${}^{\prime }$* solutions using 500 mM NaCl, knowing that such increased salt concentrations lower the overall solubility of the PEO chains in water [43,44]. In addition, we investigated a F108-N${}_{3}$-AA${}^{\prime }$ DNA sample with a 5:1 molar ratio thus doubling the amount of nucleotides in the system. Extracting the zero-frequency viscosities from our DLS-based microrheology measurements we plot the relative-to-solvent viscosity ratios for the different added salt and DNA concentrations in Figure 6.

In Figure 2, we saw that 10 % w/v F108 solutions with 100 mM added NaCl have a CMT $∼23{\;}^{{}^{\circ}}$C. Above that temperature we observed a strong upward trend in the viscosity due to the conversion of unimers into micelles until all polymer chains were packed into micelles, which was at T∼ 35 ${}^{{}^{\circ}}$C. This was followed by a steady decrease in viscosity at even higher temperatures. As expected, in the presence of 500 mM added NaCl the CMT of the F108 solution dropped to below 15 ${}^{{}^{\circ}}$C. The relative viscosity peaked around $T=27{\;}^{{}^{\circ}}$C following the same trend as in the low salt case. This behaviour is in agreement with a previous study on a similar system [50] that reports both the disappearance of large micellar clusters and the dehydration of the micelle cores. This is confirmed by the relaxation times extracted from the $g^{\left(1\right)}\left(\tau \right)$ curves we measured.

The DNA functionalised systems show again a remarkably different rheological response. Firstly, all viscosities are up to three times lower than those of the F108 solutions, in particular at higher temperatures. Furthermore, a clear observation of the CMT was not possible for any of the systems within our experimental setup. The reduced viscosity of these F108-N${}_{3}$-*AA${}^{\prime }$* samples is a direct result of the fact that azide and DNA binding leads to the aggregation and thus formation of a liquid of disconnected, transient clusters that is dominated by the viscosity of the aqueous background. A similar reduction in viscosity was found in solutions of DNA nanostars connected via linear DNA linkers [37] and in dense solutions of aggregating proteins [64].

**Figure 7:**
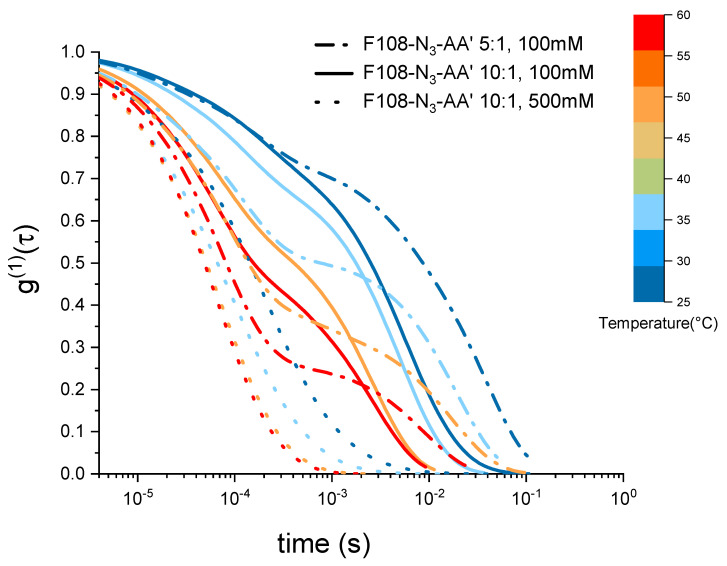
Electric field autocorrelation functions $g^{\left(1\right)}\left(\tau \right)$ of 10 % w/v F108-N${}_{3}$-*AA${}^{\prime }$* with 10:1 and with 1:5 molar ratios as function of temperature and added NaCl measured in DLS.

Interestingly, the two F108-N${}_{3}$-*AA${}^{\prime }$* samples measured for 100 mM added NaCl reveal a viscosity maximum at around $T∼27{\;}^{{}^{\circ}}$C followed by a linear decrease with increasing temperature suggesting that doubling the DNA content was not sufficient to overcome the azide driven formation of flower micelles that connect to each other. Further, we do not observe appreciable changes near the DNA melting temperature $T_{m}=48{\;}^{{}^{\circ}}$. The change in the dominance of the different interactions becomes evident in the autocorrelation curves shown in Figure 7. There the $g^{\left(1\right)}\left(\tau \right)$ curves for the 5:1 molar ratio of the F108-N${}_{3}$-*AA${}^{\prime }$* show longer relaxation times than the 10:1 sample with lower amount of possible *AA${}^{\prime }$*-DNA bonds at lower temperatures, while above the DNA melting temperature the azide–azide interaction between the free F108-N${}_{3}$ ends dominates. Hence, if all F108-N${}_{3}$ chain-ends would be terminated with DNA, the effect of the azides could be completely suppressed. Surprisingly, the presence of the higher added salt concentration seems to improve the solubility of the azide ends of the F108-N${}_{3}$ chains to the point of even influencing the micelle formation of the triblock copolymer and thus keeping the overall viscosity change relatively small across the temperature range studied. Another possible interpretation could of course be that the azide groups are zwitter-ionic in nature, although on average they appear neutral. Hence, the additional salt may screen the Coulomb interactions between them, thus preventing micelle formation or growth of larger aggregates. At this point we are not able to distinguish the two different mechanisms as these would require further analysis.

## 4. Discussion and Conclusions

In this work, we first verified the known phase diagram of the non-functionalised F108 system (Figure 1D) confirming the gradual unimer-to-micelle transition occurring upon heating using visual inspection, dynamic light scattering and DLS-based microrheology [65]. In short, for a given F108 concentration, the number of micelles increases with increasing temperature above the CMT until all unimers are converted into micelles leading to a micellar liquid for lower concentrations. This was observed as an increase of the systems viscosity followed by a subsequent decrease for steadily increasing temperatures (Figure 2B). Furthermore, our DLS measurements showed that the F108 micelles have a hydrodynamic radius $R_{H}∼12$ nm, which is close to estimates obtained from SAXS measurements [66].

Introducing azide and N${}_{3}$-DNA groups to end-functionalise the hydrophilic PEG chain-ends, we hoped to introduce binding between them at low temperatures, leading to a viscoelastic, entangled network of concatenated F108 chains. Using the thermo-reversibility and programmability of DNA we thus could tune the systems’ self-assembling behaviour [11,36]. However, our DLS and phase diagram measurements showed a completely different picture. We hypothesise that the un-reacted azide groups, whilst not chemically reactive, develop a strong hydrophobic attraction to each other that leads to a macroscopic phase separation above sufficiently high concentrations and temperatures. We propose that the N${}_{3}$ groups render the F108 chains telechelics that tend to form flower-micelles below the CMT (Figure 2C), where water is a good solvent for the PEO and PPO blocks. Above the CMT, water becomes an increasingly bad solvent for the PEO middle-block forcing an inversion of the flower micelles into micelles with a water-free PEO core while the azide ends now point outwards to connect to other PEG-azide ends forming either intra-micellar loops or bridges between different micelles. As these hydrophobic forces must be of the order of a few $k_{B}T$, both the entire F108 chains can leave a micelle and join another continuously and the azide groups can bind between micelles and break rapidly, giving rise to a transient behaviour.

Normal- and shear force measurements by Eiser et al. [67] demonstrated that telechelic chains in good solvent condition form a stretchered polymer-brush with the chain ends facing the free interface, where they form sticky flower micelles, instead of loops. These measurements confirmed the fast dynamics between transiently connected chain ends reported in literature [68,69]. Further, the group of Port reported rheology and SAXS data on the phase behaviour of mixtures of oil-in-water micro-emulsions and telechelic chains [70,71]. They found a first-order phase transition similar to ours at high enough concentrations of the telechelic chains. This phase transition was precluded by a transient sol-gel percolation line at lower telechelic concentrations. This is in agreement with our DLS-findings, namely, that both the F108-N${}_{3}$ and the partially DNA-functionalised solutions show many long relaxation times below a critical F108 concentration (here about 15 % w/v; Figure 3). Above that concentration, our F108-N${}_{3}$ solutions show a clear, thermodynamic phase transition, which was analysed by Zilman et al. as an entropic phase transition [72].

Partial functionalisation of the F108-N${}_{3}$ chain ends with DNA suppressed the macroscopic phase separation. Nevertheless, the long transient relaxation times remain present. Hence, only when all chain ends are DNA-functionalised can we suppress this behaviour and turn the system into one whose phase behaviour is dominated by the hybridisation of the hydrophilic DNA duplex below its melting temperature.

To conclude, the presence of a small, hydrophobic moiety at the end of Pluronics can lead to transient hydrogels that can be further functionalised with a small fraction of bio-molecular sensors such as DNA. Such gels can be of great practical importance in the design of affordable tissues or diagnostic tools [73], also because such transient gels are in general know to be self-healing materials. Moreover, azide groups, attached to PEO, are often used to covalently link DNA to colloid surfaces to introduce highly selective binding rules. If too many N${}_{3}$-groups remain unreacted, unwanted non-specific interactions can influence the overall behaviour of such systems, which needs to be considered in the future.

## Data Availability

All data will be made available upon request to the corresponding author.
